# P-583. Admission Diagnoses and Outcomes among Hospitalized People Living with HIV during Pre-COVID-19, COVID-19 and Post-COVID-19 Pandemic Periods

**DOI:** 10.1093/ofid/ofae631.781

**Published:** 2025-01-29

**Authors:** Thana Khawcharoenporn, Pattarapoom Panutat

**Affiliations:** Thammasat University, Bangkok, Krung Thep, Thailand; Faculty of Medicine, Thammasat University, Pathumthani, Pathum Thani, Thailand

## Abstract

**Background:**

Coronavirus disease 2019 (COVID-19) has affected human immunodeficiency virus (HIV) care in many ways. However, impacts on admission diagnoses and outcomes among hospitalized people living with HIV (PLHIV) have not been evaluated.

**Methods:**

A retrospective cohort study was conducted among hospitalized PLHIV at a Thai tertiary care center during the 3 periods, Period 1: Pre-COVID-19 (September 2017-March 2020), Period 2: COVID-19 (April 2020-September 2022, and Period 3: Post-COVID-19 (October 2022-October 2023). Characteristics, admission diagnoses and outcomes were compared between PLHIV admitted during these 3 periods. Factors associated with hospital mortality were determined.

**Results:**

Of the 310 PLHIV included, 117, 125 and 68 were admitted during Period 1, 2 and 3, respectively and 115 (37%) were newly diagnosed HIV on admission. Median CD4 cell counts and proportions of those with antiretroviral therapy (ART) adherence rates of ≥95% at admission were different between the periods [(206, 97 and 138 cells/mm^3^ and 97%, 89% and 100% in Period 1, 2, and 3 (P=0.02 and P=0.06)]. Of the 310 PLHIV, admission diagnoses were non-AIDS-related (62%) and AIDS-related (38%). Most of the non-AIDS-related diagnoses were infections (40%) while opportunistic infections were the most common for AIDS-related diagnoses (88%). The types of admission diagnoses were not different between the 3 periods. Among 62 PLHIV who developed in-hospital complications, 47% and 39% had nosocomial infections and drug-related complications, respectively. Hospital mortality rates were 10%, 13% and 16% during Period 1, 2, and 3 (P=0.80). By multivariable analysis, intensive care unit admission, underlying malignancy, monthly income less than $USD 400, and admission CD4 count less than 50 cells/mm^3^ were independently associated with hospital mortality.

**Conclusion:**

Although admission during COVID-19 pandemic period was not associated with increased mortality, we observed the impact of the pandemic on the lower CD4 cell count and ART adherence at admission among hospitalized PLHIV. Interventions to improve early care engagement, ART adherence, and close monitoring for those with identified mortality risks are needed for better HIV care, especially during pandemics.

**Disclosures:**

**All Authors**: No reported disclosures

